# The Hemoglobin Bjgb From *Bradyrhizobium diazoefficiens* Controls NO Homeostasis in Soybean Nodules to Protect Symbiotic Nitrogen Fixation

**DOI:** 10.3389/fmicb.2019.02915

**Published:** 2020-01-10

**Authors:** Ana Salas, Germán Tortosa, Alba Hidalgo-García, Antonio Delgado, Eulogio J. Bedmar, David J. Richardson, Andrew J. Gates, María J. Delgado

**Affiliations:** ^1^Department of Soil Microbiology and Symbiotic Systems, Estación Experimental del Zaidín, Consejo Superior de Investigaciones Científicas, Granada, Spain; ^2^Laboratory of Stable Isotopes Biogeochemistry, Instituto Andaluz de Ciencias de la Tierra, Consejo Superior de Investigaciones Científicas, Granada, Spain; ^3^School of Biological Sciences, University of East Anglia, Norwich, United Kingdom

**Keywords:** bacterial hemoglobins, nitrogen fixation, nitrogenase, nitric oxide, ^15^N isotope, soybean nodules

## Abstract

Legume-rhizobia symbiotic associations have beneficial effects on food security and nutrition, health and climate change. Hypoxia induced by flooding produces nitric oxide (NO) in nodules from soybean plants cultivated in nitrate-containing soils. As NO is a strong inhibitor of nitrogenase expression and activity, this negatively impacts symbiotic nitrogen fixation in soybean and limits crop production. In *Bradyrhizobium diazoefficiens*, denitrification is the main process involved in NO formation by soybean flooded nodules. In addition to denitrification, nitrate assimilation is another source of NO in free-living *B. diazoefficiens* cells and a single domain hemoglobin (Bjgb) has been shown to have a role in NO detoxification during nitrate-dependent growth. However, the involvement of Bjgb in protecting nitrogenase against NO in soybean nodules remains unclear. In this work, we have investigated the effect of inoculation of soybean plants with a *bjgb* mutant on biological nitrogen fixation. By analyzing the proportion of N in shoots derived from N_2_-fixation using the ^15^N isotope dilution technique, we found that plants inoculated with the *bjgb* mutant strain had higher tolerance to flooding than those inoculated with the parental strain. Similarly, reduction of nitrogenase activity and *nifH* expression by flooding was less pronounced in *bjgb* than in WT nodules. These beneficial effects are probably due to the reduction of NO accumulation in *bjgb* flooded nodules compared to the wild-type nodules. This decrease is caused by an induction of expression and activity of the denitrifying NO reductase enzyme in *bjgb* bacteroids. As *bjgb* deficiency promotes NO-tolerance, the negative effect of NO on nitrogenase is partially prevented and thus demonstrates that inoculation of soybean plants with the *B. diazoefficiens bjgb* mutant confers protection of symbiotic nitrogen fixation during flooding.

## Introduction

Legumes have the unique ability to establish a dinitrogen (N_2_)-fixing symbiotic association with soil bacteria collectively termed rhizobia. Consequently, inoculation of legumes with rhizobia can substantially reduce the dependency on synthetic nitrogen fertilizers. This has several advantages, including mitigating greenhouse gas (GHG) emissions as well as protecting ground water from N-oxyanion contamination while improving soil fertility. In this context, a better understanding of the microorganisms associated with legume crops would contribute to improve food security and to reduce climate change. Following invasion of the root plant cells through a signaling exchange between bacteria and plant, rhizobia stop dividing and undergo differentiation into nitrogen-fixing bacteroids. In the bacteroid, nitrogenase is the key enzyme that reduces atmospheric N_2_ into biologically useful forms in a process called “Biological N Fixation” (reviewed by Sprent, 2009; Udvardi and Poole, 2013; Poole et al., 2018). Several studies have reported that nitrogenase is inhibited by nitric oxide (NO) (Kato et al., 2010; Sanchez et al., 2010). This highly reactive gas also contributes to the ozone layer depletion and has multiples roles in diverse physiological processes in living organisms. At low concentrations (nmolar levels) NO acts as signaling molecule, while at higher concentrations (μmolar levels) it is a pathological or toxic agent (Toledo and Augusto, 2012). In plants, NO is involved in many processes essential for growth and development. This signaling molecule is also implicated in the response of plants to many abiotic stresses (hypoxia, salinity, heavy metals, among others) (Corpas and Barroso, 2015; Domingos et al., 2015). During plant-pathogen interactions, NO also plays a key role in the hypersensitive response during plant defense (Thalineau et al., 2016). Interestingly, NO is also formed during the legume-rhizobia symbiotic interactions. In the root nodules, both the bacterial and plant partners are involved in NO production (reviewed by Hichri et al., 2015; Berger et al., 2018). From the plant perspective, NO is formed by the plant nitrate reductase (NR), nitrite (NO_2_^–^): NO reductase activity associated to the mitochondrial electron transport chain (ETC), and the NO synthase (NOS)-like activity (see Figure 3). In addition to plant sources, bacteroidal denitrification and particularly the periplasmic nitrate and nitrite reductases (Nap, and NirK, respectively) have been reported to be also involved in NO formation in root nodules (Sanchez et al., 2010; Horchani et al., 2011) (see Figure 3).

In the legume-rhizobia symbiosis, NO has an essential role during the establishment of the symbiotic interaction favoring nodule formation and development (Boscari et al., 2013). However, in mature nitrogen-fixing nodules, NO negatively affects nitrogenase expression and activity (Kato et al., 2010; Sanchez et al., 2010). This effect is more significant in response to hypoxia caused by flooding where an induction of NO formation occurs (Sanchez et al., 2010). Given the evidence for NO production in root nodules, the presence of NO-detoxification systems is crucial for maintaining a low steady-state intracellular NO concentration to support an efficient symbiosis. In this context, non-symbiotic and symbiotic plant hemoglobins have been reported to be involved in NO detoxification in nodules (Berger et al., 2018). From the bacterial perspective, several systems have been proposed to detoxify NO anaerobically such as the nitric oxide reductase (Nor) from denitrifiers, cytochrome *c* respiratory nitrite reductase (NrfA) from nitrate-ammonifying bacteria, or flavorubredoxin (NorVW), among others (reviewed by Stern and Zhu, 2014; Torres et al., 2016). Here, NO is detoxified by reduction to either ammonia or nitrous oxide (N_2_O). Bacterial hemoglobins (Hbs) are also a large family of important and well-characterized proteins for aerobic NO detoxification in bacteria. There are three main classes of Hbs: flavohemoglobins (fHb), single domain hemoglobins (sdHb), and truncated hemoglobins (tHb) (for a review see Poole, 2005; Stern and Zhu, 2014; Gell, 2018). FHbs, such as Hmp from *Escherichia coli*, consist of three domains: an N-terminal domain with a heme *b*-type cofactor, a central FAD binding domain and a C-terminal NADP-binding domain (Poole, 2005). In the presence of oxygen, reduced heme from Hmp catalyzes the reaction between NO and O_2_ producing nitrate by either NO denitrosylase mechanism or O_2_ dioxygenase process (Gardner et al., 1998; Hausladen et al., 2001; Gardner, 2005). Under anaerobiosis, Hmp has reductase activity and it is capable to reduce NO to N_2_O (Kim et al., 1999). The sdHbs, such as Cgb from *Campylobacter jejuni*, also have a role in NO detoxification. They contain the hemoglobin domain, but they do not possess the oxidoreductase domain and the FAD-containing domain (for a review see Tinajero-Trejo et al., 2013). TrHbs, such as HbN from *Mycobacterium tuberculosis*, lacks the oxidoreductase domain and the FAD-containing domain and its hemoglobin domain is 20–40 residues shorter than classical sdHbs (Poole, 2005). In the legume root nodules, the denitrifying Nor rhizobial enzyme has been shown to be involved in NO removal (Sanchez et al., 2010; Blanquet et al., 2015). In addition to Nor, a combined role for *Ensifer meliloti* fHb (Hmp), and NnrS_1_ and NnrS_2_ proteins in NO degradation has been reported in *Medicago truncatula* nodules (Cam et al., 2012; Meilhoc et al., 2013; Blanquet et al., 2015).

*Bradyrhizobium diazoefficiens* is a Gram-negative soil bacterium that fixes N_2_ during symbiotic interaction with soybean plants (*Glycine max*). This bacterium is also able to denitrify under free-living conditions or inside the root nodules (reviewed by Bedmar et al., 2005, 2013; Torres et al., 2016). In *B. diazoefficiens*, denitrification reactions are catalyzed by four enzymes that reduce nitrate (Nap), nitrite (NirK), nitric oxide (Nor), and nitrous oxide (Nos), respectively. These enzymes are encoded by *napEDABC* (Delgado et al., 2003), *nirK* (Velasco et al., 2001), *norCBQD* (Mesa et al., 2002), and *nosRZDYFLX* (Velasco et al., 2004) genes. The denitrification process by *B. diazoefficiens* bacteroids is proposed to be the main driver for NO formation in soybean nodules in response to flooding conditions (Sanchez et al., 2010). In addition to denitrification, a coordinated nitrate assimilation and NO detoxification system, encoded by the *narK*-*bjgb*-*flp*-*nasC* operon, is also involved in NO homeostasis within free-living cells. This cluster codes for a putative single domain hemoglobin (Bjgb), the assimilatory nitrate reductase (NasC), a nitrate/nitrite transporter (NarK) and a FAD-dependent NAD(P)H oxidoreductase (Flp). Bjgb mitigates the NO produced by NasC as by-product of nitrate/nitrite assimilation (Cabrera et al., 2016). Thus, a role for Bjgb in protecting *B. diazoefficiens* free-living cells from nitrosative stress has been proposed (Cabrera et al., 2016). However, the function of *B. diazoefficiens* Bjgb in soybean nodules remains unclear. The aim of this research is to analyze the role of Bjgb from *B. diazoefficiens* in the response of symbiotic nitrogen fixation to flooding as well as in the NO homeostasis in soybean nodules.

## Materials and Methods

### Bacterial Strains and Growth Conditions

*Bradyrhizobium diazoefficiens* USDA 110 (WT) (United States Department of Agriculture, Beltsville, MD, United States), and a *bjgb* deletion mutant (named 4001 strain), which was previously constructed by Cabrera et al. (2016), were used in this study. *B. diazoefficiens* strains were grown under aerobic conditions at 30°C in peptone-salts-yeast extract (PSY) medium added with 0.1% (w/v) L-arabinose (Regensburger and Hennecke, 1983). For inocula preparation, cells were collected by centrifugation at 8000 *g* for 10 min, washed twice and cultured aerobically for 48 h at 30°C in Bergersen minimal medium (Bergersen, 1977) where glycerol was substituted by 10 mM succinate as carbon source and L-glutamate was replaced by 10 mM KNO_3_ as sole N-source. Chloramphenicol was added to the cultures at 20 μg ml^–1^.

### Plant Growth Conditions

Soybean (*G. max* L. Merr., cv. Williams) seeds were surface-sterilized with ethanol for 5 min, immersed in H_2_O_2_ (30%, v/v) for 15 min, and finally washed with sterile distilled water. Then, seeds were germinated in 1% agar petri-dishes (8–9 seeds each) and incubated in darkness at 30°C for 72 h. Seedlings were sowed in autoclaved Leonard jars which contained vermiculite (Trung and Yoshida, 1983). Two soybean plants per jar were inoculated at sowing with 1 ml of a single bacterial strain (approx. 10^8^ cells ml^–1^) and overlaid with autoclaved perlite. Plants were transferred to a plant growth chamber for 35 days (16–8 h day/night cycle, day/night temperatures of 26–22°C and photosynthesis photon flux density of 128–148 μmol photons m^–2^ s^–1^). Plants were cultivated using a mineral solution (Rigaud and Puppo, 1975) with or without 4 mM KNO_3_. Treatment of plants with 4 mM KNO_3_ does not inhibit nodule formation or nitrogenase activity as previously reported by Mesa et al. (2004). After growth for 28 days, a set of plants were kept under flooding for 7 days by immersing them to 1 cm above substrate level applying mineral solution as described previously (Sanchez et al., 2010). For determination of the plant N content acquired from biological N_2_ fixation, plants were watered with 4 mM ^15^N-labeled KNO_3_ (Potassium nitrate-^15^N; 5 atom% ^15^N; Cat. #CS01-185_272; Campro Scientific GmbH). Nodules were collected from 35-day-old plants. Plant physiological parameters were determined per plant: nodule number per plant (NNP), nodule dry weight per plant (NDWP), and plant dry weight (PDW).

### Total Nitrogen and Nitrogen Derived From Biological N_2_ Fixation

These analyses were carried out as previously described (Sanchez et al., 2011b). Plants were oven-dried and then weighed and grounded in an IKA A 11 basic analytical mill (Rose Scientific Ltd., Alberta, Canada). For total nitrogen (TN) and ^15^N enrichment (δ^15^N), subsamples of 3 mg were analyzed with an elemental analyzer (EA1500 NC, Carlo Erba, Milan, Italy) combined to isotope-ratio mass spectrometer (Delta Plus XL, ThermoQuest, Bremen, Germany). The general precision of analyses for δ^15^N was ± 0.1‰. The stable composition was shown as δ^15^N values per mil: δ^15^N (‰) = (*R*_sample_/*R*_standard_ − 1) × 1000, where *R* = ^15^N/^14^N. Commercial N_2_ was the internal standard for the nitrogen isotopic analyses. δ^15^N data for all samples were standardized against internationally accepted reference materials (IAEA N1, δ^15^N = +0.4‰, IAEA N2, δ^15^N = +20.3‰ and USGS32 δ^15^N = +174.5 vs. AIR). The proportion of N derived from the atmosphere (%Ndfa) was calculated by following the formula: %Nfda = 100 × [1 − (*A*/*B*)], where *A* = Atom% ^15^N excess in inoculated plants, *B* = Atom% ^15^N excess in uninoculated plants. Atom% ^15^N excess = atom% ^15^N in labeled treatment – atom% ^15^N in non-labeled treatment. Atom% ^15^N was calculated as: δ^15^N (‰) × 100. To calculate the atom% ^15^N excess, a set of plants was grown only under N_2_-fixing conditions to obtain the atom% ^15^N of the non-labeled treatment. The fixed-nitrogen content (FN) was calculated as: FN = (%Ndfa × TN)/100.

### Acetylene Reduction Activity

**FIGURE 1:**
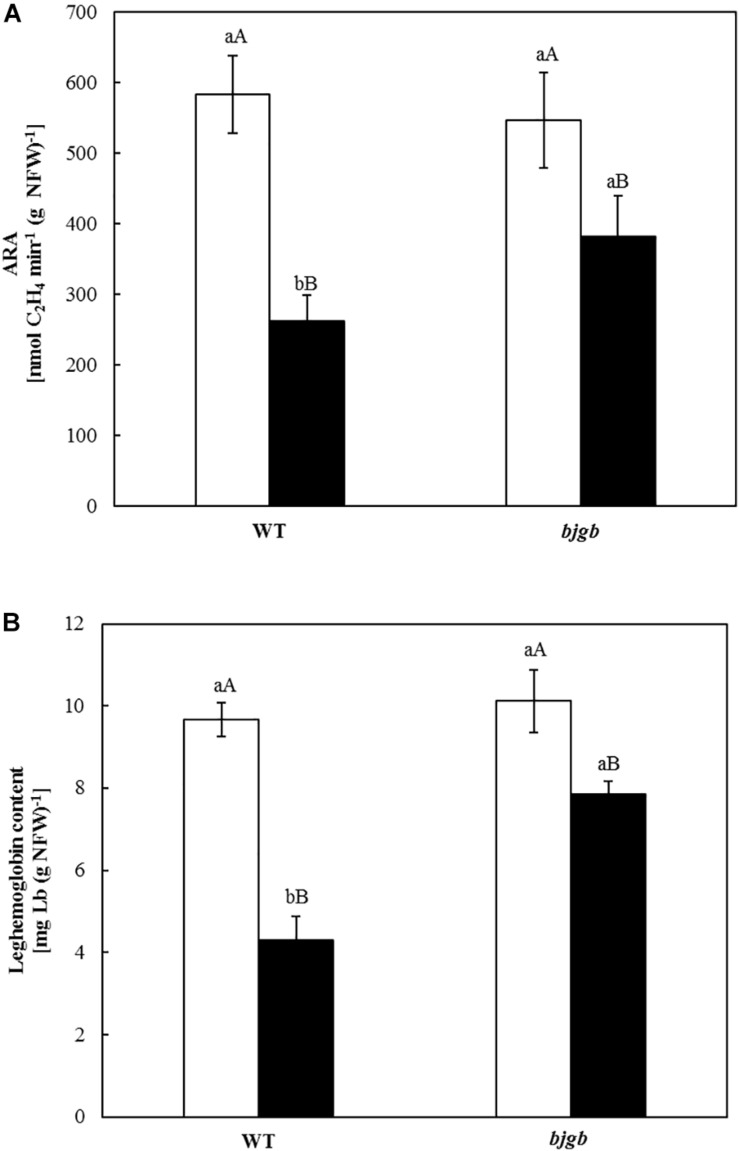
Acetylene reduction activity (ARA) **(A)** and Leghemoglobin (Lb) content of soybean nodules **(B)**. Plants were inoculated with *B. diazoefficiens* USDA 110 (WT) or 4001 (*bjgb* mutant) strains. Nodules were isolated from non-flooded plants (white bars) or plants subjected to flooding conditions for 7 days (black bars). Data are means with standard deviations from two independent experiments assayed by using six replicates. In individual graphs, bars within the same treatment marked with the same lower-case letter, and bars within the same strain followed by the same capital letter, are not significantly different as determined by the ANOVA test at *P* ≤ 0.05.

Acetylene reduction activity (ARA) was analyzed with fresh detached nodules from plants provided with mineral solution containing 4 mM KNO_3_. About 20 nodules per replica were placed in 20-ml headspace vials (SUPELCO^®^) containing 100 μl mineral solution added with 4 mM KNO_3_. Tubes were sealed, filled with 1 ml of pure acetylene and incubated at 30°C. After incubation for 2 and 4 h, gas samples (0.5 ml) were extracted from the tubes for ethylene analysis. A Hewlett-Packard model 5890 gas chromatograph (Agilent Technologies, S.L., Madrid) with a flame ionization detector and a molecular sieve 5A (60–80 mesh) column (180 cm × 0.32 cm) (Agilent Technologies, S.L.) was used. N_2_ at 60 ml min^–1^ was used as carrier gas. Temperatures of oven, injector, and detector were 60, 90, and 110°C, respectively. Ethylene concentration in each sample was calculated from standards of 2% (v/v) ethylene. Acetylene reduction rate was calculated by measuring the increase of ethylene (C_2_H_4_) production inside the vials headspace determined in the lineal range (0, 2, and 4 h of incubation) (Supplementary Figure S1) and calculated as: Δ C_2_H_4_ nmol (4–2 h)/Δ time (4–2 h). Results presented in Figure 1A were expressed as: [nmol C_2_H_4_ min^–1^ (g NFW) ^–1^] where NFW = nodule fresh weight.

### Leghemoglobin Content

Leghemoglobin (Lb) was quantified in nodules from plants provided with mineral solution containing 4 mM KNO_3_ following the protocol described by Sanchez et al. (2010). Fluorimetric detection of Lb was analyzed in a Shimadzu (Shimadzu Scientific Instruments, Kyoto, Japan) spectrophotofluorometer with a mercury-xenon lamp and a RF-549 red-sensitive photomultiplier. The excitation wavelength was 405 nm and the emission monochromator adjustment was 650 nm. Heme protein concentration was proportional to the difference in fluorescence between heated and unheated samples and therefore it was indicative of the leghemoglobin concentration in the samples. Lb content in each sample was calculated using bovine hemoglobin as standard. Lb content was expressed as: [mg Lb (g NFW) ^–1^].

### Transcript Levels Determination

Nodules were detached from roots, frozen in liquid nitrogen and stored at −80°C until further use. For RNA isolation, nodules were distributed in 2-ml Eppendorf tubes and were disrupted and homogenized by using a TissueLyser (Mixer Mill MM 301; Retsch GmbH, Haan, Germany) (three times for 50 s at 30 Hertz) with a tungsten carbide bead (3 mm; Qiagen). Then, RNA was isolated by following the hot phenol-extraction protocol described elsewhere (Babst et al., 1996). RNA integrity was confirmed by agarose gel electrophoresis. Precipitated RNA was treated with DNaseI amplification grade (Invitrogen). Next, RNA samples were cleaned up with RNeasy Mini spin columns (Qiagen). Genomic DNA contamination was checked by PCR using fixN4_For and fixN4_Rev primers (Supplementary Table S1). Then, 2 μg of total RNA was reverse transcribed to cDNA employing random hexamers and SuperScript II reverse transcriptase (Invitrogen) following the supplier’s instructions. For quantitative reverse transcriptase-polymerase chain reaction (qRT-PCR) analyses, an iQTM5 Optical System (Bio-Rad, Foster City, CA, United States) was used. Each PCR reaction (with a total volume of 19 μl) was performed using 9.5 μl iQ^TM^ SYBR Green Supermix (Bio-Rad), 2 μM (final concentration) of individual primers (Supplementary Table S1) and proper dilutions of different cDNA samples. The PCR program included an initial denaturation and *Taq* activation step of 5 min at 95°C, followed by 40 cycles of 15 s at 95°C, 45 s at 60°C, and 45 s at 72°C. PCR reactions were performed in triplicate. The formation of specific PCR products was checked by melting curve analyses. Expression of the gene encoding the 16S rRNA was used as reference for normalization (primers 16S_qRT_For and 16S_qRT _Rev; Supplementary Table S1). Relative changes were calculated by using the Pfaffl method (Pfaffl, 2001). Expression of *narK* in WT flooded nodules was determined relative to that observed in WT non-flooded nodules. For *nifH* and *norC* expression, values were calculated relative to the levels of *nifH* or *norC* expression in WT non-flooded nodules. The expression levels given as fold-change for both the WT and *bjgb* mutant were compared with those from WT under non-flooding conditions.

### Detection of NO in Nodules

Nitric oxide was detected in fresh detached nodules (about five nodules) from plants provided with mineral solution added with 4 mM KNO_3_. For NO scavenger treatment, nodules were incubated for 1 h in the dark at 30°C in a solution containing 2-[4-carboxyphenyl]-4,4,5,5-tetramethylimidazoline-1-oxyl-3-oxide (c-PTIO) (Sigma-Aldrich) at a final concentration of 3 mM. Then, NO was detected by dipping the nodules in 7 μM of 4,5-diaminofluorescein diacetate (DAF-2 DA) solution (Genaxxon bioscience) for 2 h in the dark at 30°C, while control nodules were incubated in distilled water under the same conditions. The relative fluorescence units of the DAF-2 DA solution were determined using a fluorometer (MD-5020, Photon Technology International, Birmingham, NJ, United States) with 495 nm excitation and 515 nm emission wavelength (2 nm band width). Nodular NO accumulation was expressed as relative fluorescence units per nodule dry weight (NDW): [RFU (mg NDW)^–1^].

### Nitrous Oxide Production by Nodules

N_2_O was measured in fresh detached nodules from plants provided with mineral solution supplemented with 4 mM KNO_3_. Essentially, detached nodules from roots were placed in 20-ml headspace vials (SUPELCO^®^) (about 20 nodules) provided with 100 μl (for nodules incubated under non-flooding conditions) or 5 ml (to maintain nodules under flooding treatment) of mineral solution with 4 mM KNO_3_. Acetylene 10% (v/v) was injected into each tube in order to inhibit nitrous oxide reductase (Yoshinari and Knowles, 1976). Samples were incubated for 6 h at 30°C before measuring N_2_O production. Then, gaseous aliquots (1 ml) were taken from the headspace using a gas-tight syringe and they were manually injected into an HP 4890D gas chromatograph with an electron capture detector and a Porapak Q 80/100 MESH (8 ft) packed column. N_2_ at 28 ml min^–1^ was the carrier gas. The injector, column and detector temperatures were 125, 60, and 375°C, respectively. Concentrations of N_2_O in the samples were determined employing 2% (v/v) N_2_O as standard. Total N_2_O concentration was calculated considering N_2_O in headspace and dissolved N_2_O using Bunsen water solubility coefficient (54.4% at 25°C). For each replicate, N_2_O flux was recorded after 4 and 6 h of incubation, which was the lineal range of N_2_O emission. The N_2_O emission flux was calculated as: Δ N_2_O molar concentration (6–4 h)/Δ time increase (6–4 h). Results were expressed as: [nmol N_2_O h^–1^ (g NDW) ^–1^].

### Statistical Analyses

The total number of replicates for each assay is shown in each figure and table. For each parameter, descriptive statistical analyses (media and standard deviation) were calculated and data were checked for normal distribution and homoscedasticity. The analyses of variance (ANOVA) within treatments were performed using the *post hoc* Tukey–Kramer test (*P* ≤ 0.05). For inferential statistical analyses, GNU-PSPP open-source software v0.9.0^1^ was used.

## Results

### Loss of *B. diazoefficiens* Bjgb Confers Tolerance of Soybean Symbiotic Nitrogen Fixation to Flooding

After 35 days of growth, NNP, NDWP, and PDW were analyzed (Table 1). As previously reported (Mesa et al., 2004), the presence of nitrate in the nutrient solution did not inhibit NNP or NDWP in plants inoculated with either the WT or the *bjgb* mutant (Table 1). Flooding treatment did not significantly alter NNP in plants grown either in the presence or in the absence of nitrate, independently of the strain used as inoculum (Table 1). On the contrary, flooding provoked a significant inhibition of NDWP of plants cultivated with or without nitrate. Interestingly, those plants inoculated with the *bjgb* mutant and grown with nitrate showed higher NDWP in response to flooding (about 30%) than plants that were inoculated with the WT (Table 1). As expected, the presence of nitrate increased PDW compared to non-nitrate treated plants. As shown in Table 1, in the absence of nitrate, flooding did not significantly affect PDW in plants inoculated with any of the strains. However, in nitrate-treated plants, flooding provoked a negative effect on PDW that was only significant (about 28%) in those plants inoculated with the WT strain but not in those inoculated with the *bjgb* mutant (Table 1). As control of symbiotic nitrogen fixation, a set of uninoculated plants were included in the analyses. After 35 days growth, nodulation did not take place in uninoculated plants and very low levels of PDW were observed in those plants grown without nitrate (Table 1). In plants that were only nitrate dependent, flooding decreased PDW about 36% (Table 1).

**TABLE 1:** Nodule number per plant (NNP), nodule dry weight per plant (NDWP), and plant dry weight (PDW) of plants inoculated with *B. diazoefficiens* USDA 110 (WT) or 4001 (*bjgb* mutant) strains.

		**− Nitrate**			**+ Nitrate**		
			
**Strain**	**Treatment**	**NNP**	**NDWP (g plant^–1^)**	**PDW (g)**	**NNP**	**NDWP (g plant^–1^)**	**PDW (g)**
USDA 110 (WT)	−F	53 ± 11 aA	0.174 ± 0.046 aA	1.95 ± 0.83 aA	57 ± 8 aA	0.161 ± 0.012 bA	3.40 ± 0.51 aA
	+F	41 ± 12 aA	0.088 ± 0.020 aB	1.66 ± 0.39 aA	48 ± 11 aA	0.057 ± 0.007 bB	2.45 ± 0.26 aB
4001 (*bjgb*)	−F	49 ± 14 aA	0.169 ± 0.048 aA	1.93 ± 0.70 aA	61 ± 9 aA	0.192 ± 0.011 aA	3.61 ± 0.64 aA
	+F	45 ± 9 aA	0.096 ± 0.010 aB	1.49 ± 0.27 aA	52 ± 13 aA	0.081 ± 0.010 aB	2.74 ± 0.96 aA
Uninoculated	−F	–	–	0.65 ± 0.07 bA	–	–	3.25 ± 0.53 aA
	+F	–	–	0.66 ± 0.08 bA	–	–	2.09 ± 0.49 aB

*Nodules were isolated from plants grown in the absence (−Nitrate) or the presence of 4 mM KNO_3_ (+Nitrate). Seven days before harvesting plants were subjected (+F) or not (−F) to flooding conditions. Values (means ± standard deviations) in a column within the same treatment marked with the same lower-case letter, and values in a column within the same strain followed by the same capital letter, are not significantly different as determined by the ANOVA test at *P* ≤ 0.05 (*n* = 6).*

As is indicated in Table 2, flooding stress provoked a significant decrease in the TN content of shoots from inoculated plants grown with 4 mM KNO_3_ (Table 2). Nevertheless, plants inoculated with *bjgb* showed an increase of about 12% in TN compared to plants where the WT was used as inoculum (Table 2). In uninoculated plants cultured with nitrate, flooding did not affect TN (Table 2). In order to differentiate between N acquired from N_2_-fixation or nitrate assimilation, we used the ^15^N isotope dilution technique that allowed us to determine the proportion of N derived from the atmosphere (Ndfa%) in plants that were cultivated with 4 mM ^15^N-labeled KNO_3_. Here, flooding decreased %Ndfa of plants, being this reduction significantly higher in plants inoculated with the WT compared to those inoculated with the *bjgb* mutant (55% vs. 30%). Consequently, the content of fixed nitrogen (FN) of flooded plants inoculated with *bjgb* was about 47% higher than that of flooded plants inoculated with the WT (Table 2). These results clearly indicate that inoculation of the plants with the *bjgb* mutant confers tolerance of symbiotic nitrogen fixation to flooding. By using the ^15^N isotope dilution technique, we also calculated the % of ^15^N atom in excess (atom% ^15^N excess) of the shoots. As shown in Table 2, ^15^N excess in uninoculated plants was significantly higher compared to inoculated plants and no effect of flooding in ^15^N excess of uninoculated plants was perceived. However, in plants inoculated either with the WT or *bjgb* mutant, flooding increased the % of ^15^N excess. These results indicate that soybean nitrogen fixation is more sensitive to flooding than nitrate assimilation supporting previous findings (Bacanamwo and Purcell, 1999; Sanchez et al., 2011b).

**TABLE 2:** Atom ^15^N excess, proportion of nitrogen derived from the atmosphere (%Ndfa), total nitrogen content (TN) and fixed-nitrogen content (FN) of shoot tissue of uninoculated plants or plants inoculated with *B. diazoefficiens* USDA 110 (WT) or 4001 (*bjgb* mutant) strains.

**Strain**	**Treatment**	**Atom ^15^N excess (%)**	**Ndfa (%)**	**TN (mg g^–1^)**	**FN (mg g^–1^)**
USDA 110 (WT)	−F	2.66 ± 0.30 bB	39.32 ± 6.77 aA	27.97 ± 2.84 aA	10.98 ± 2.03 aA
	+F	3.40 ± 0.45 bA	17.87 ± 5.41 bB	14.94 ± 0.85 bB	2.63 ± 0.64 bB
4001 (*bjgb*)	−F	2.67 ± 0.55 bA	39.16 ± 12.52 aA	26.20 ± 2.55 aA	10.39 ± 2.01 aA
	+F	3.11 ± 0.84 bA	27.45 ± 4.70 aA	16.94 ± 0.90 aB	4.94 ± 0.90 aB
Uninoculated	−F	4.39 ± 0.77 aA	–	12.53 ± 1.41 bA	–
	+F	4.44 ± 0.12 aA	–	13.48 ± 1.54 bA	–

*Plants grown in the presence of 4 mM K ^15^NO_3_ were subjected (+F) or not (−F) to flooding conditions for 7 days. Values (means ± standard deviations) in a column within the same treatment marked with the same lower-case letter, and values in a column within the same strain followed by the same capital letter, are not significantly different as determined by the ANOVA test at *P* ≤ 0.05 (*n* = 6).*

In order to confirm the flooding effect on N_2_ fixation, nitrogenase activity was measured by determining ARA in nodules incubated for 2 and 4 h (Supplementary Figure S1). Values for C_2_H_4_ produced in nmol per min and g NFW (Figure 1A) showed a decrease in ARA (about 55%) for WT nodules isolated from plants submitted to flooding compared to non-flooded nodules. However, nitrogenase activity only decreased about 30% in *bjgb* nodules from flooded plants compared to non-flooded *bjgb* nodules (Figure 1A). Functionality of the nodules was also estimated by measuring the Lb content (Figure 1B). Consistent with ARA determinations, flooding provoked a smaller decrease of Lb content in nodules produced by the *bjgb* mutant (about 22%) compared to that observed in WT nodules (about 55%).

Next, we tested the effect of deleting *bjgb* on the expression of the *nifH* gene which is responsible for the synthesis of the Fe-protein from nitrogenase complex (Table 3). Transcript levels for *nifH* were examined in WT or *bjgb* nodules by qRT-PCR. As observed in Table 3, *nifH* expression was not significantly affected in the nodules induced by the *bjgb* mutant (−1.31 fold-change) compared to WT nodules, both collected from non-flooded plants. Flooding decreased *nifH* expression by ∼16-fold in WT nodules compared to that observed in WT non-flooded nodules. However, *nifH* mRNA levels decreased by ∼10-fold in *bjgb* flooded nodules compared to those observed in WT non-flooded nodules.

**TABLE 3:** Expression of *narK*, *nifH*, and *norC* in nodules measured by qRT-PCR.

		**Relative amount of transcript**	
		**(fold-change)**	
		
**Strain**	**Gene**	**No. flooding**	**Flooding**
USDA 110 (WT)	*narK*	1.00 ± 0.00	+ 11.18 ± 1.85
4001 (*bjgb*)	*narK*	nd	nd
USDA 110 (WT)	*nifH*	1.00 ± 0.00	−16.33 ± 3.07
4001 (*bjgb*)	*nifH*	−1.31 ± 0.28	−10.61 ± 1.91
USDA 110 (WT)	*norC*	1.00 ± 0.00	+ 67.53 ± 6.86
4001 (*bjgb*)	*norC*	+ 1.12 ± 0.28	+ 89.16 ± 8.59

*Nodules were harvested from plants inoculated with *B. diazoefficiens* USDA 110 (WT) or 4001 (*bjgb* mutant) strains. Plants were grown in the presence of 4 mM KNO_3_ and subjected or not to flooding conditions for 7 days. Data are means ± standard deviations from there independent RNA samples assayed by using three replicates. nd, not determined, +, increased expression, −, decreased expression.*

In order to ascribe the effect of *bjgb* inoculation to the presence of Bjgb in the nodules, we checked the expression of the *bjgb* gene by analyzing transcript levels of *narK*, the lead gene of the *narK*-*bjgb*-*flp*-*nasC* transcriptional unit (Cabrera et al., 2016) (Table 3). As shown in Table 3, *narK* expression increased ∼11-fold in flooded nodules compared to non-flooded nodules collected from plants inoculated with the WT strain.

### Loss of *B. diazoefficiens* Bjgb Reduces NO Levels in Soybean Nodules in Response to Flooding

**FIGURE 2:**
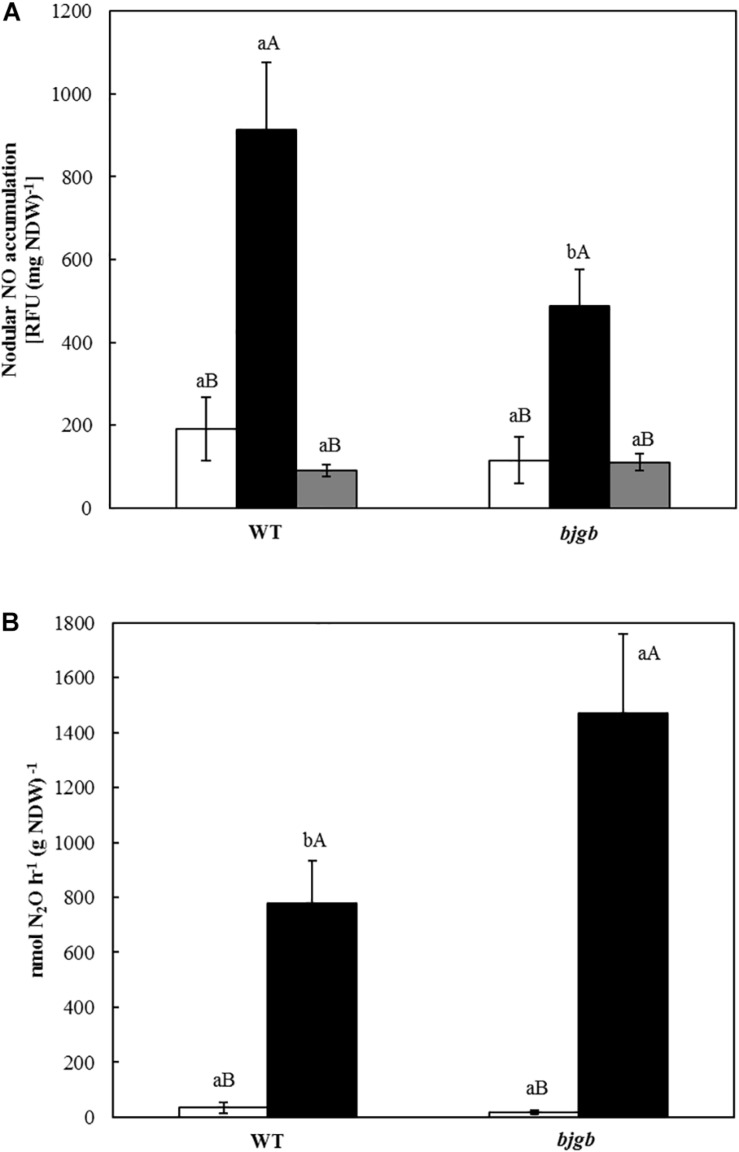
Nitric oxide (NO) detection **(A)** and N_2_O production **(B)** by soybean nodules. Plants were inoculated with *B. diazoefficiens* USDA 110 (WT) or 4001 (*bjgb* mutant) strains. Nodules were isolated from non-flooded plants (white bars) or plants subjected to flooding conditions for 7 days (black bars). In A, flooded nodules were incubated with 3 mM c-PTIO (gray bars). Data are means with standard deviations from two independent experiments assayed by using six replicates. In individual graphs, bars within the same treatment marked with the same lower-case letter, and bars within the same strain followed by the same capital letter, are not significantly different as determined by the ANOVA test at *P* ≤ 0.05.

The contribution of *B. diazoefficiens* Bjgb in NO homeostasis in nodules was investigated by analyzing the capacity to accumulate NO as well as to produce N_2_O, the product of the nitric oxide reductase (Nor). To perform these experiments, we used nodules from plants grown under the conditions that induced NO and N_2_O accumulation, as previously reported (Sanchez et al., 2010; Tortosa et al., 2015). Thus, nodules were collected from soybean plants inoculated with *B. diazoefficiens* USDA 110 (WT) or 4001 (*bjgb* mutant) strains, cultivated with 4 mM nitrate and submitted or not to flooding conditions for 7 days before harvesting. Free NO was detected by using the DAF-2DA specific fluorescent probe. As shown in Figure 2A, very low levels of NO were observed in WT and *bjgb* nodules of non-flooded plants. However, flooding significantly induced NO formation in WT nodules confirming previous results (Sanchez et al., 2010). This induction was also observed in nodules from plants inoculated with the *bjgb* mutant that accumulated approximately 2-fold less NO than WT nodules in response to flooding conditions (Figure 2A). In order to prove that the increase of fluorescence perceived in flooded nodules was caused by NO, WT, and *bjgb* flooded nodules were incubated with a NO scavenger (c-PTIO). After incubation of the nodules with c-PTIO, nodular NO accumulation was significantly reduced (Figure 2A), indicating that the fluorescence signal was mostly due to NO production.

We also analyzed Nor activity by measuring N_2_O production by soybean nodules using gas chromatography. While non-flooded nodules produced by WT or *bjgb* strains showed basal levels of N_2_O, flooding conditions induced N_2_O production in *B. diazoefficiens* WT nodules, consistent with previous observations (Tortosa et al., 2015). Interestingly, nodules produced by the *bjgb* mutant accumulated about twofold more N_2_O than WT nodules in response to flooding (Figure 2B). The lower NO levels as well as higher N_2_O production capacity observed in the nodules from the *bjgb* mutant compared to WT levels (Figures 2A,B), suggest that Nor activity that reduces NO to N_2_O is induced in *bjgb* bacteroids. In order to establish if the differences of Nor activity observed between WT and *bjgb* bacteroids could be explained by changes in *nor* gene expression, *norC* transcripts were measured by performing qRT-PCR analyses. RNA was isolated from nodules harvested from soybean plants inoculated with *B. diazoefficiens* WT or *bjgb* mutant strains. In nodules from plants that were not subjected to flooding, *bjgb* mutation did not significantly affect *norC* expression compared to wild-type (+1.12 fold-change). Flooding provoked a notable increase of *norC* expression in either WT or *bjgb* flooded-nodules compared to WT non-flooded nodules (+67.53 and +89.16 fold-change, respectively). Interestingly, the induction of *norC* expression by flooding was about 33% higher in *bjgb* nodules than in WT nodules (Table 3).

## Discussion

Several studies have reported that NO production in soybean nodules is induced by nitrate and hypoxia that is promoted by flooding conditions (Meakin et al., 2007; Sanchez et al., 2010). This molecule is a potent inhibitor of nitrogenase activity and expression (Kato et al., 2010; Sanchez et al., 2010). In nodules, NO can also bind Lb contributing to the formation of nitrosyl-leghemoglobin (LbNO) complexes that have a major role in detoxifying reactive nitrogen species (Sanchez et al., 2010). It is also well established that the denitrification pathway in *B. diazoefficiens* is the major source of NO and N_2_O in soybean nodules (Sanchez et al., 2010; Tortosa et al., 2015). Previous experiments revealed that in soybean plants inoculated with a *B. diazoefficiens napA* null strain, where denitrification is inhibited, basal levels of NO and N_2_O were still detected in nodules (Sanchez et al., 2010; Tortosa et al., 2015). These observations suggested that, in addition to denitrification, other mechanisms give rise to NO and N_2_O in nodules. The recently identified hemoglobin Bjgb is one such system which has been reported to be involved in NO detoxification under free living conditions (Cabrera et al., 2011, 2016; Sanchez et al., 2011a), however, its function inside the nodules is unknown. In this work, the role of Bjgb in symbiotic nitrogen fixation and NO homeostasis in the *B. diazoefficiens*–*G. max* symbiosis has been investigated by inoculating plants with a *bjgb* mutant. In order to induce NO formation in the nodules, soybean plants were grown in the presence of KNO_3_ (4 mM) and 7 days before harvesting they were subjected to flooding, as previously reported (Mesa et al., 2004; Meakin et al., 2007; Sanchez et al., 2010). This nitrate concentration was selected based in a previous work (Mesa et al., 2004) where *G. max* L. Merr., cv. Williams inoculated with *B. diazoefficiens* USDA 110 was grown in the presence of 0, 1, 2, 3, 4, 5, and 6 mM KNO_3_. Mesa et al. (2004) found that 4 mM KNO_3_ resulted in the induction of bacteroidal denitrification by measuring N_2_O formation in nodules, and this concentration did not inhibit either nodule formation or nitrogenase activity. Since the effect of flooding on NNP was similar independently of the strain used for inoculation, the increase in NDWP observed in plants where *bjgb* was used as inoculum is probably due to a higher individual nodule weight. This observation suggests that *bjgb* mutant has a beneficial effect on nodule development and growth rather than on nodule number formation.

The impact of flooding on nitrogen fixation was further investigated by calculating the amount of FN in shoots employing the ^15^N isotope dilution method. Consistent with previous observations (Sanchez et al., 2011b), this approach was determinant to demonstrate the negative effect of flooding on nitrogen fixation in plants grown in the presence of nitrate. The decrease of FN provoked by flooding was significantly less pronounced in plants that were inoculated with the *bjgb* mutant, indicating that the loss of Bjgb confers tolerance of N_2_ fixation to flooding. These findings were confirmed by analysis of nitrogenase activity (ARA) and levels of leghemoglobin (estimation of nodule functionality). As reported previously, flooding significantly inhibited ARA and Lb levels of WT nodules (Sanchez et al., 2010). However, the negative effect of flooding on ARA and Lb levels was smaller in nodules from plants inoculated with the *bjgb* mutant compared to WT nodules.

In contrast to our findings, inoculation of *M. truncatula* plants with an *E. meliloti* strain lacking the flavohemoglobin (Hmp) strongly inhibited ARA and provoked nodule senescence (Cam et al., 2012), as compared to those inoculated with the WT. The authors attributed this effect to an increase in the NO levels observed in the nodules produced by the *hmp* mutant which negatively affected nitrogen fixation and increased root nodule senescence. Contrary to the higher NO levels observed in *M. truncatula* nodules induced by the *E. meliloti hmp* mutant compared to those produced by the WT (Cam et al., 2012), our results showed that NO formation was significantly lower in *bjgb* than in WT nodules in response to flooding. The apparent differences observed regarding the involvement of the *E. meliloti hmp* and *B. diazoefficiens bgjb* mutants in N_2_-fixation and nodule NO formation could be due to the different plant growth conditions used by Cam et al. (2012) and in this work. While Cam et al. cultured *M. truncatula* in nitrogen−free medium, in the present work soybean plants were grown with nitrate and subjected to flooding for 7 days. In *B. diazoefficiens*, denitrification accounts for about 90% of NO present in flooded soybean nodules (Sanchez et al., 2010), while in *M. truncatula* nodules, *E. meliloti* produces only ∼35% of NO detected (Cam et al., 2012). The contradictory observations found between the roles of *E. meliloti* Hmp and *B. diazoefficiens* Bjgb might be also due to metabolic differences in their nitrate-reducing pathways. While *B. diazoefficiens* is a complete denitrifier which grows anoxically by nitrate respiration, *E. meliloti* is unable to respire nitrate under anoxic conditions (reviewed by Torres et al., 2016). Furthermore, it might also be possible that the nature of nodule-type supports different mechanisms of dealing with the nitrosative stress, considering that indeterminate nodules (*M. truncatula*) are characterized by a persistent meristem and a continuous growth, while determinate nodules (*G. max*) are characterized by a not persistent meristem and a limited growth potential.

The decreased levels of NO produced by *bjgb* nodules could explain the tolerance of nitrogen fixation to flooding observed in plants inoculated with the *bjgb* mutant. In *B. diazoefficiens* there are two main processes involved in NO formation, which are denitrification and nitrate assimilation. Under low oxygen nitrate-dependent free-living conditions, pathways for both respiratory denitrification and nitrate assimilation are active to promote bacterial survival. In fact, both respiratory (Nap) and assimilatory (NasC) nitrate reductases contribute similarly to the total activity. Under these conditions, a *bjgb* mutant showed substantial growth inhibition compared to WT cells suggesting a NO detoxifying role for Bjgb (Cabrera et al., 2016). However, under symbiotic conditions, where growth is not needed, the contribution of assimilatory nitrate reduction in the bacteroids is only ∼10% (Sanchez et al., 2010). Consequently, it may be possible that low NO concentrations arising from nitrate assimilation in the bacteroids cytoplasm does not present toxicity in nodules. These observations may also explain that, in contrast to the reported role in NO detoxification during nitrate-dependent anaerobic growth, Bjgb would not be directly involved in NO detoxification inside nodules and thus may instead act as an NO-buffer.

The assimilatory nitrate reductase (NasC) is encoded by the *narK-bjgb-flp-nasC* operon that also contains the gene encoding the sdHb (Bjgb). Cabrera et al. (2016) reported that NO produced by NasC in the cytoplasm acts as signal molecule which activates expression of the denitrifying *nor* genes. In this context, it has been recently demonstrated that NO is the signaling molecule that induces *nor* genes in *B. diazoefficiens* (Bueno et al., 2017). Under free-living conditions, expression of the respiratory Nor was significantly up-regulated in a *bjgb* mutant relative to WT, probably due to increased intracellular NO levels that arise during assimilatory nitrate reduction (Cabrera et al., 2016). In soybean nodules, NO produced by the periplasmic denitrifying enzyme NirK is the main source of NO, and Nor the principal NO removal protein (Sanchez et al., 2010). It might be possible that, as it has been demonstrated in free-living cells (Cabrera et al., 2016), NO produced in the cytoplasm from nitrate assimilation is increased in bacteroids induced by the *bjgb* mutant. Then, this molecule would act in the cytoplasm as a signal that induces *nor* genes expression. In fact, induction of *norC* expression by flooding was greater in the *bjgb* nodules than in WT nodules. Furthermore, analysis of Nor activity showed a higher N_2_O formation capacity in *bjgb* nodules compared to those produced by the parental strain.

**FIGURE 3:**
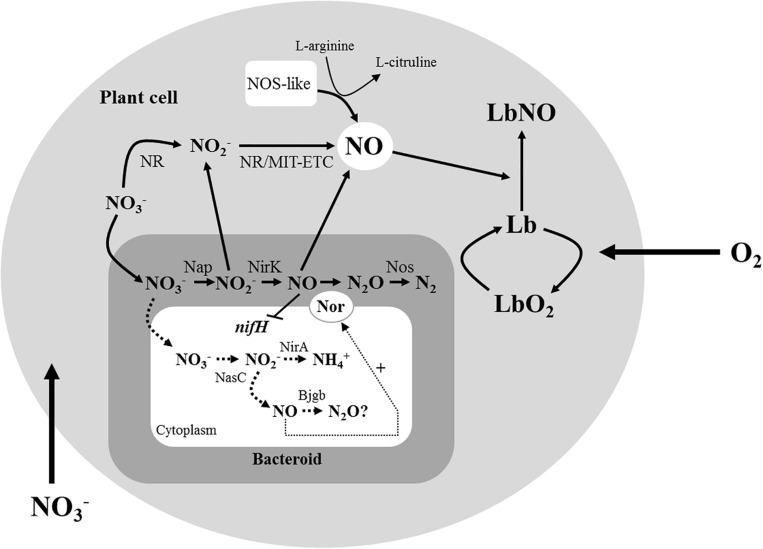
Proposed role of Bjgb in soybean nodules, alongside well-characterized sources of NO in nodules. The large gray circle represents the plant cell, and the small gray square represents the bacteroid where the periplasm is shown in gray and the cytoplasm is shown in white. Adapted from Torres et al. (2016). In addition to the reported plant sources of NO in legume nodules, denitrification in the bacteroids also contributes to the formation of this molecule. During this process, NO_2_^–^ and NO produced by Nap and NirK, respectively, can bind Lb producing LbNO complexes. It has also been reported that NO is an inhibitor of *nifH* expression. Results from this work suggest that in addition to denitrification, assimilatory nitrate reduction by NasC might be another source of NO in nodules. NO produced in the bacteroid cytoplasm by this system would act as a signal molecule to activate *nor* genes. The ability of Bjgb to bind NO *in vitro* or to reduce NO to N_2_O *in vivo* (indicated with a question mark) is under investigation.

It has been previously reported that soybean plants inoculated with a *B. diazoefficiens nirK* mutant, whose nodules do not produce NO from denitrification, were more tolerant to flooding than plants inoculated with the WT strain (Sanchez et al., 2011b). Similarly, results obtained in this work suggest that inoculation with the *bjgb* mutant partially diminished the negative effect of flooding on N_2_ fixation observed in WT-inoculated plants. This advantage is probably due to the increased capacity of *bjgb* deficient nodules to induce expression of *nor* genes, whereby the gene product removes the NO produced by denitrification. The decreased levels of NO observed in *bjgb* nodules compared to WT nodules in response to flooding lead us to suggest that *B. diazoefficiens* Bjgb, instead of functioning as a direct NO-detoxifying protein in the nodules, it would contribute indirectly by modulating cytoplasmic NO levels, the signaling molecule required for induction of the denitrifying nitric oxide reductase enzyme, which is the major protein involved in NO removal in soybean nodules (see Figure 3). In conclusion, this work reveals a strategy for nitrogenase protection and consequently for efficient symbiotic nitrogen fixation that requires the modulation of NO levels in root nodules by the microsymbiont. Therefore, using rhizobia strains that modulate NO levels in nodules is an important practice that would enhance legume production and promote sustainable agriculture. From this perspective, the contribution of *bjgb* mutation is positive for nitrogenase protection. However, elevated N_2_O production resulting from increased NO reduction results in release of a potent and stable GHG that has a negative environmental impact and may contribute to climate change.

## Data Availability Statement

All datasets generated for this study are included in the article/Supplementary Material.

## Author Contributions

AS, AG, and MD conceived and designed the study, analyzed the results, and wrote the manuscript. AS, GT, AH-G, and AD performed the experiments. DR and EB critically revised the manuscript. All authors read and approved the final version of the manuscript.

## Conflict of Interest

The authors declare that the research was conducted in the absence of any commercial or financial relationships that could be construed as a potential conflict of interest.

## Footnotes

1 https://www.gnu.org/software/pspp/

## Supplementary Material

FIGURE S1 Ethylene emission by soybean nodules. Plants were inoculated with *B. diazoefficiens* USDA 110 (WT) (squares) or 4001 (*bjgb* mutant) (circles) strains. Nodules were isolated from non-flooded plants (white symbols) or plants subjected to flooding conditions for 7 days (black symbols). Data are means with standard deviations from two independent experiments assayed by using six replicates.

TABLE S1 Primers used for qRT-PCR analyses.

## Abbreviations

ARA: acetylene reduction activity
Bjgb: *Bradyrhizobium diazoefficiens* hemoglobin
fHb: flavohemoglobin
Flp: flavoprotein
FN: fixed nitrogen
Hbs: hemoglobins
Lb: leghemoglobin
N_2_O: nitrous oxide
Nap: periplasmic respiratory nitrate redutase
NarK: nitrate/nitrite transporter
NasC: assimilatory nitrate reductase
Ndfa: nitrogen derived from the atmosphere
NDW: nodule dried weight
NDWP: nodule dry weight per plant
NFW: nodule fresh weight
NifH: Fe-protein of the nitrogenase complex
NirK: copper-dependent respiratory nitrite reductase
nmol: nanomol
NNP: nodule number per plant
NO: nitric oxide
Nor: nitric oxide reductase
PDW: plant dry weight
TN: total nitrogen
WT: wild-type.
