# Supplementary figures and images for: The Hemoglobin Bjgb From Bradyrhizobium diazoefficiens Controls NO Homeostasis in Soybean Nodules to Protect Symbiotic Nitrogen Fixation
Source: Front Microbiol. 2020 Jan 10;10:2915. doi: 10.3389/fmicb.2019.02915 (PMC6965051; doi:10.3389/fmicb.2019.02915)

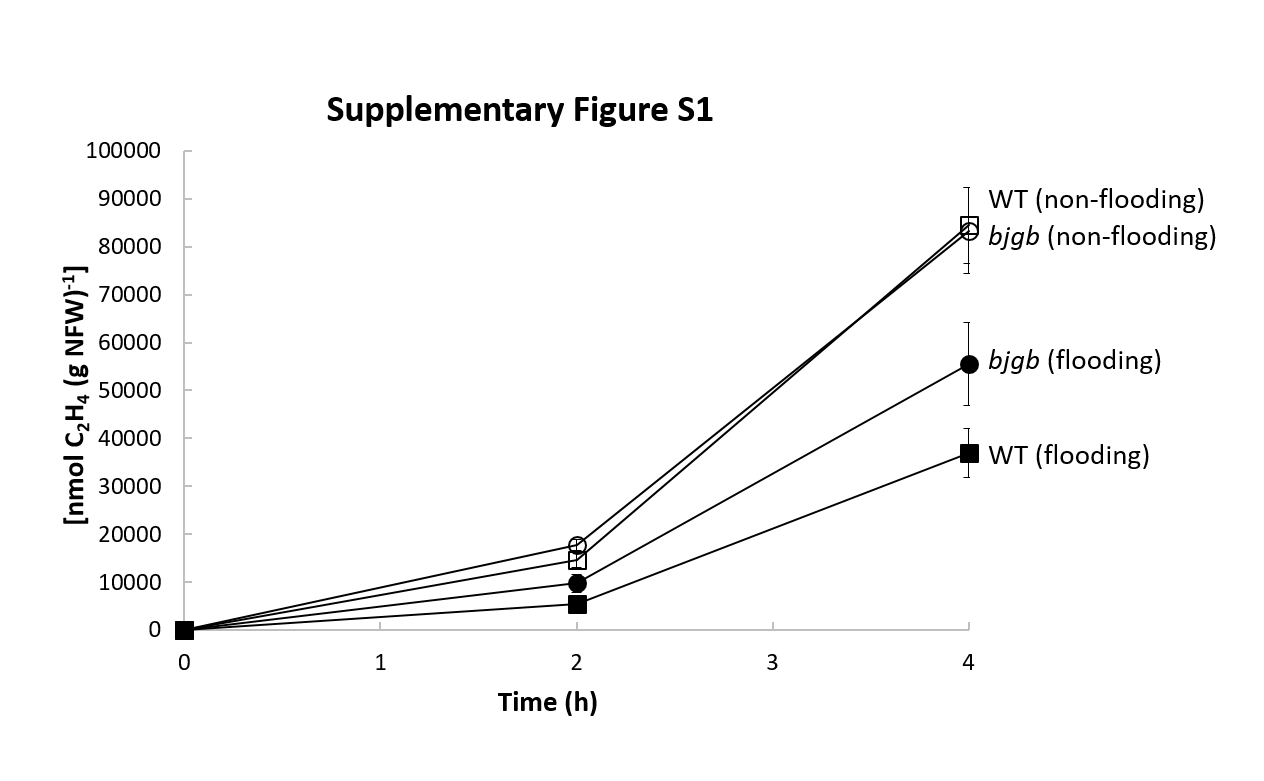

Supplement: FIGURE S1 — Ethylene emission by soybean nodules. Plants were inoculated with B. diazoefficiens USDA 110 (WT) (squares) or 4001 (bjgb mutant) (circles) strains. Nodules were isolated from non-flooded plants (white symbols) or plants subjected to flooding conditions for 7 days (black symbols). Data are means with standard deviations from two independent experiments assayed by using six replicates. [file Image_1.tif]
