# Supplementary material for: The Hemoglobin Bjgb From Bradyrhizobium diazoefficiens Controls NO Homeostasis in Soybean Nodules to Protect Symbiotic Nitrogen Fixation
Source: Front Microbiol. 2020 Jan 10;10:2915. doi: 10.3389/fmicb.2019.02915 (PMC6965051; doi:10.3389/fmicb.2019.02915)
Supplement: TABLE S1 — Primers used for qRT-PCR analyses. [file Table_1.DOCX]

| **Table S1.**  Primers used for qRT-PCR analyses | | |
| --- | --- | --- |
| **Primer** | **DNA sequence (5’ → 3’)** | **Reference** |
|  |  |  |
| fixN4_For | CGGGATCCCGACTCCTATCCGGTCGAGGAC | ([Mesa et al., 2005](#_ENREF_1)) |
| fixN4_Rev | CGGAATTCCGGGTCATGGATTTGGAGATGG |  |
|  |  |  |
| nifH_For | CGGCAGACCGACAAGGAA | ([Sánchez et al., 2010](#_ENREF_2)) |
| nifH_Rev | ATCAGTTGAGTGCCAAGCTTCTT |  |
|  |  |  |
| narK_For | TATCGGCTTCTCGGTCTGG | This work |
| narK_Rev | GCTGGTCGGTGGTGTAATG |  |
|  |  |  |
| norC_3_For | GCAGATGCCGCAGTTCAAC | ([Torres et al., 2014](#_ENREF_3)) |
| norC_3_Rev | TGATCGTGCTCACCCATTG |  |
|  |  |  |
| 16S_qRT_For | GCAGGCTTAACACATGCAAGTC | ([Torres et al., 2017](#_ENREF_4)) |
| 16S_ qRT _Rev | AGGTACGTTCCCACGCGTTACTC |  |

**References**

Mesa, S., Ucurum, Z., Hennecke, H., and Fischer, H.M. (2005). Transcription activation in vitro by the *Bradyrhizobium japonicum* regulatory protein FixK_2_. *J. Bacteriol.* 187**,** 3329-3338. doi: 10.1128/JB.187.10.3329-3338.2005.

Sánchez, C., Gates, A.J., Meakin, G.E., Uchiumi, T., Girard, L., Richardson, D.J., et al. (2010). Production of nitric oxide and nitrosylleghemoglobin complexes in soybean nodules in response to flooding. *Mol. Plant Microbe Interact.* 23**,** 702-711. doi: 10.1094/MPMI-23-5-0702.

Torres, M.J., Argandoña, M., Vargas, C., Bedmar, E.J., Fischer, H.M., Mesa, S., et al. (2014). The global response regulator RegR controls expression of denitrification genes in *Bradyrhizobium japonicum*. *PLoS One* 9**,** e99011. doi: 10.1371/journal.pone.0099011.

Torres, M.J., Bueno, E., Jiménez-Leiva, A., Cabrera, J.J., Bedmar, E.J., Mesa, S., et al. (2017). FixK_2_ Is the Main Transcriptional Activator of *Bradyrhizobium diazoefficiens nosRZDYFLX* Genes in Response to Low Oxygen. *Front. Microbiol.* 8**,** 1621. doi: 10.3389/fmicb.2017.01621.
